# The Influence of SARS-CoV-2 Infection on Lipid Metabolism—The Potential Use of Lipid-Lowering Agents in COVID-19 Management

**DOI:** 10.3390/biomedicines10092320

**Published:** 2022-09-18

**Authors:** Klaudia Kowalska, Zofia Sabatowska, Joanna Forycka, Ewelina Młynarska, Beata Franczyk, Jacek Rysz

**Affiliations:** Department of Nephrology, Hypertension and Family Medicine, Medical University of Lodz, ul. Żeromskiego 113, 90-549 Lodz, Poland

**Keywords:** lipoproteins, COVID-19, lipid metabolism, HDL, LDL, TG, lipoproteins as biomarkers, lipid-lowering therapies, statins

## Abstract

Several studies have indicated lipid metabolism alterations during COVID-19 infection, specifically a decrease in high-density lipoprotein (HDL) and low-density lipoprotein (LDL) concentrations and an increase in triglyceride (TG) levels during the infection. However, a decline in triglycerides can also be observed in critical cases. A direct correlation can be observed between a decrease in serum cholesterol, HDL-C, LDL-C and TGs, and the severity of the disease; these laboratory findings can serve as potential markers for patient outcomes. The transmission of coronavirus increases proportionally with rising levels of cholesterol in the cell membrane. This is due to the fact that cholesterol increases the number of viral entry spots and the concentration of angiotensin-converting enzyme 2 (ACE2) receptor, crucial for viral penetration. Studies have found that lower HDL-C levels correspond with a higher susceptibility to SARS-CoV-2 infection and infections in general, while higher HDL-C levels were related to a lower risk of developing them. However, extremely high HDL-C levels in serum increase the risk of infectious diseases and is associated with a higher risk of cardiovascular events. Low HDL-C levels are already accepted as a marker for risk stratification in critical illnesses, and higher HDL-C levels prior to the infection is associated with a lower risk of death in older patients. The correlation between LDL-C levels and disease severity is still unclear. However, TG levels were significantly higher in non-surviving severe patients compared to those that survived; therefore, elevated TG-C levels in COVID-19 patients may be considered an indicator of uncontrolled inflammation and an increased risk of death.

## 1. Introduction

In December 2019 international attention was drawn to Wuhan, China, where healthcare professionals reported an increasing number of cases of severe pneumonia with no clear etiology [1,2]. The following year, on January 7, a novel coronavirus was isolated from patients in Wuhan and given the name: severe acute respiratory syndrome coronavirus 2 (SARS-CoV-2) by the International Committee on Taxonomy of Viruses (ICTV) on February 11 [1]. As the World Health Organization (WHO) announced the pandemic on March 11, 2020, COVID-19 became the third pandemic associated with coronaviruses (CoVs) since the beginning of the twenty-first century, preceded by the SARS and Middle East Respiratory Syndrome (MERS) pandemics [3]. Since then, there have been 603,711,760 confirmed cases of the disease, including 6 484 136 deaths according to the WHO coronavirus dashboard. The COVID-19 pandemic has been a challenge to medical professionals and the disease has also become a subject of extensive research. It has also become a burden to public health, and economic and social stability all over the globe, putting people’s lives on hold for almost a year until the first vaccine was authorized for emergency use by the US Food and Drug Administration (FDA) [4]. Given the reluctance of some members of the public to be vaccinated, difficulties in vaccine distribution, and new variants of SARS-CoV-2, the pandemic control measures as well as the disease itself will remain a challenge in the future [5,6,7,8].

Although SARS-CoV-2 infection principally affects the lungs, its manifestation spreads beyond the respiratory tract [9]. The main receptor participating in SARS-CoV-2 invading host cells is the angiotensin-converting enzyme 2 (ACE2) receptor [2]. As the ACE2 protein is also expressed in the gastrointestinal tract, renal tissues, heart, liver, and blood vessels, there is a wide range of COVID-19 symptoms [8]. Alterations in lipid profiles have also been reported due to SARS-CoV-2 infection, with a decline in serum cholesterol, low-density lipoprotein cholesterol (LDL-C) and high-density lipoprotein cholesterol (HDL-C) concentrations [10,11]. Lipids are basic cell components with various functions ranging from a structural one, a signaling molecule and a central energy storage [12]. The human plasma lipoprotein fractions and their main functions are summarized in Table 1. Lipids also play a significant role in viral endocytosis, replication, and exocytosis [13]. Cholesterol regulates the entry of the SARS-CoV-2 virus into host cells as cholesterol-enriched rafts are specific platforms that enable viral endocytosis [14]. Viral replication inside the host cell disrupts lipid metabolism and lipid resources as SARS-CoV-2 comprises a bilayer lipid envelope [12,13]. In addition, inflammation and cytokine storms have a negative impact on the natural lipid biosynthesis pathways [10]. Moreover, lipids are believed to have an immunomodulatory role [14]. HDLs, besides their anti-atherosclerotic properties, have anti-inflammatory and antioxidant functions [14].

Studies have shown significant changes occur to cholesterol, HDL-C and LDL-C concentrations in patients with CoV infections [15]. In this review we aim to summarize current knowledge about the effects of SARS-CoV-2 infection on lipid metabolism, establish the role of lipoproteins as prognostic markers, and discuss the benefits of lipid-lowering therapies in patients with the coronavirus disease.

## 2. Membrane and Serum Cholesterol and Their Roles during Infection

CoVs, like other enveloped viruses, enter the host’s cells via endocytosis by interacting with lipid rafts within the cell membrane [16]. Lipid rafts are subdivisions of the cell membrane rich in glycosphingolipids and cholesterol, and contain a plethora of molecules, such as dynamin, caveolin and clathrin, which might be important in viral infiltration [17,18,19]. Cholesterol levels directly affect the permeability of lipid rafts; hence, a higher concentration of cholesterol in the membrane facilitates endocytosis. Conversely, lower membrane-bound cholesterol reduces viral penetration [19]. This correlation is also affected by the fact that cholesterol significantly increases the concentration of ACE2 receptors in the cell membrane [19,20,21]. According to an in vitro study by Glende et al., the cholesterol-rich microdomains provide a platform to facilitate effective interaction between the S protein and the cellular ACE2 receptor, and the role of cholesterol in this infection can be attributed to the S protein and is not affected by other coronavirus proteins [22]. Wang et al. found that in an in vitro model of cholesterol-loaded cells, the number and apparent diameter of monosialotetrahexosylganglioside (GM1) lipid rafts (viral endocytic entry points of ACE2) increased. The study also showed that cholesterol can simultaneously transport ACE2 receptors to the endocytic entry point [23]. The ACE2 receptor has been proven crucial in mediating viral penetration into the host’s cells [20]. In conclusion, the transmission of CoVs increases proportionally with rising levels of cholesterol in the cell membrane [21,24]. The relationship implies that using cholesterol-lowering drugs could be appropriate in COVID-19 treatment, as they reduce cholesterol concentrations [12,13] and improve viral clearance from the blood [13], followed by their immunomodulatory, anti-inflammatory, infection preventing and antithrombotic effects [25,26].

During the infection cholesterol levels tend to decrease, directly correlating with the severity of symptoms and leading to hypolipidemia [11], although an exact mechanism explaining this occurrence has not yet been identified [27]. Such a tendency suggests that cholesterol levels could serve as an additional indicator for COVID-19 disease severity [27].

Intriguing reports on the effect of cholesterol metabolites on SARS-CoV-2 were presented by Marcello et al. in their paper on 27-hydoxycholesterol (27OHC), a side-chain cholesterol oxidation product [28]. In their in vitro study, they demonstrated an inhibitory effect of 27OHC on SARS-CoV-2 replication. There was also a significant decrease in the 27OHC serum levels of COVID-positive patients compared to the control group, reaching a 50% reduction in severe COVID-19 cases. Opposite findings were reported by Ghzaiel et al. in their study on 7-ketocholesterol (7KC), one of the earliest cholesterol oxidation products [29]. According to this study, 7KC, regarding its pro-inflammatory and pro-oxidant properties, may be a pathophysiological factor in the acute respiratory distress syndrome in COVID-19. Consequently, high concentrations of 7KC promote a more severe course of the disease and even its fatal outcome.

However, in the literature low cholesterol is known to be a risk factor for poor prognosis, not only in the COVID-19, but in other critical diseases such as sepsis. In a study conducted by Xu et al., patients with sepsis had significantly lower levels of plasma total cholesterol, HDL-C, and LDL-C compared to non-septic patients [30]. In septic patients, cholesterol levels reflected and correlated with the intensity of inflammation, and therefore the severity of the disease. HDL-C levels had the strongest correlation with IL-6 and procalcitonin levels, as well as sequential organ failure assessment [30]. HDLs are thought to play a protective role in sepsis as alterations in HDL metabolism, such as a decrease in lecithin-cholesterol acyl transferase activity and concentration, cholesteryl transfer protein activity, or an increase in phospholipid transfer activity protein and endothelial lipase activity, occur early after the development of sepsis [31]. Because of its early decrease and high stability, HDL-C is considered to be an independent predictive marker of adverse outcomes and survival in severe sepsis [32].

## 3. HDL Molecules, Their Pleiotropic Features and Their Role during Infection

HDL exhibits anti-atherosclerotic, anti-inflammatory, antiapoptotic and antithrombotic properties [33]. HDLs improve endothelial function by decreasing the expression of adhesion molecules on endothelial cells, which reduces the infiltration of white blood cells and stimulates the synthesis of nitric oxide that promotes vasodilation and counteracts inflammation [34,35]. Moreover, the major proteins that HDL consists of, ApoA-I and ApoA-II, are reported to be major antioxidant agents [33]. The antithrombotic properties of HDLs are a result of promoting prostacyclin production by endothelial cells, and down-regulating endothelial tissue factor expression [36].

The pleiotropic effects of HDLs apply directly to the immune system and host’s defense mechanisms against pathogens [37]. HDLs transport acute-phase proteins associated with inflammation and component system regulation [38]. HDL particles interact with crucial cells of the immune system, such as macrophages, dendritic cells, megakaryocytes, T-cells, and B-cells, and therefore regulate immune signaling [38]. Lipoproteins, with HDLs in the forefront, have been proven to bind and inactivate a major component in Gram-negative bacteria membranes: lipopolysaccharide (LPS), and are therefore a part of the innate immune response [15]. Regarding viral infections, HDLs may mediate the neutralization of DNA and RNA viruses as ApoA-1 has the ability to interfere with viral entry into the host cell [39]. Studies suggest that HDLs may also promote direct inactivation of viruses and inhibit viral and host cell fusion [40].

HDL particles are believed to be constantly remodeled in response to physiological or pathophysiological stimuli due to their exposure to lipid transfer proteins, lipases and lipid exchanges with other cells by cholesterol transporters [15]. Inflammation is one of the conditions that strongly regulates HDL composition, function and HDL-C levels [38]. Lower levels of HDL-C were observed in viral diseases such as acquired immunodeficiency syndrome (AIDS) [41] and hepatitis B virus (HBV) infection [42]. Alterations in lipid profiles with an HDL-C decrease were observed in critically ill patients and those with sepsis [15,43,44,45]. There were intriguing differences between in the lipid profiles detected in patients with septic shock and trauma patients in the intensive care unit (ICU) with low HDL levels in septic shock and no alteration in trauma [46].

Many studies have shown decreases in HDL-C levels in COVID-19 patients in comparison to the HDL serum levels in healthy ones, which is presented in Table 2 [47,48,49,50]. Moreover, there was an inverse relationship between the severity of the disease and HDL-C levels [47,48,51]. Low HDL and ApoA-1 levels during the infection correlate with higher levels of inflammatory markers, such as C-reactive protein (CRP) and interleukin-6 (IL-6), and also correspond with higher mortality rates [45,49]. Thus, a decrease in HDL-C levels in COVID-19 patients may be a promising predictor of disease severity and mortality [48].

Several potential mechanisms underpinning the decrease in HDL-C levels during COVID-19 infection have been proposed. Low HDL-C levels following inflammation have been attributed to a decrease in reverse cholesterol transportation and an increase in secretory phospholipase serum amyloid A (SAA), as SAA-enriched HDL are cleared more rapidly from circulation [52]. Severe inflammation causes leakage of lipoproteins and apolipoproteins into the capillaries [53]. Moreover, SARS-CoV-2 infection causes liver disfunction in almost half of cases [53]. Severe COVID-19 is considered to be SAR-CoV-2-induced sepsis, characterized by an increased release of pro-inflammatory cytokines, which decrease the synthesis and secretion of apolipoproteins [54]. Lower HDL-C levels decrease its anti-inflammatory activities, leading to further cytokine production and a reduction of HDL-C levels. In vitro studies have shown that the virus can bind to HDL particles using the spike protein of the virus [55]; however, the importance of this discovery remains unclear.

Apart from changes in HDL-C levels during COVID-19, the structure and functions of HDLs are also modified [56]. According to this analysis, ApoA-1 and paraoxonase-1 (PON-1) were less abundant, yet SAA and alpha-1 antitrypsin contents were higher [56]. However, these findings are not unique to COVID-19 as they also occur during other infections. HDLs from SARS-CoV-2-infected patients showed less protection to endothelial cells stimulated by tumor necrosis factor-α (TNFα), so HDL-related inhibition of apoptosis was blunted [56].

Not only is the decrease in HDL levels during the infection considered a promising marker for the severity of the disease; HDL-C levels before the infection may also be used a predictor [47,48]. A clinical observational study found that lower HDL-C levels corresponded with a higher susceptibility to SARS-CoV-2 infection and infections in general, while higher HDL-C levels were related to a lower risk of developing SARS-CoV-2 infection [57,58,59,60]. However, extremely high HDL-C levels in serum increased the risk of infectious diseases and was associated with a higher risk of cardiovascular events [61].

## 4. LDL Structure, Function and Plasma Concentrations during COVID-19 Infection

LDLs transport most of the cholesterol contained in the circulatory system [62]. One molecule of LDL contains one apolipoprotein, ApoB-100 [59]. Small dense LDLs are associated with hypertriglyceridemia, obesity, and resistance to insulin, their percentage is higher during infectious diseases [62]. They are considered more pro-atherogenic due to their longer retention in circulation, ease of entry into the arterial walls and being more susceptible to oxidation than larger ones [62]. LDLs act as a transporter for hydrophobic molecules, primarily cholesterol ester in a hydrophilic environment, and have the capability of neutralizing lipopolysaccharides which presents an abundance of pleiotropic properties [15,63].

Studies have shown that LDL-C levels decrease significantly during infections with varying etiology, for example, such a correlation is especially evident in dengue infections, where LDL levels directly correlate with severity [64,65]. Similarly, patients with COVID-19 present with low LDL levels according to studies by Qin et al. [27] and other examinations [11,66]. Interesting research has been conducted by Tanaka et al. in Paris, where lipoprotein levels were measured in patients admitted to the surgical ICU [67]. This study proved that all COVID-19 positive patients had low LDL-C levels; however, no evidence supporting a connection to infection outcomes was found. In addition, the correlation between the severity of the disease and low LDL-C levels was also observed by Wei et al. [11]. Curiously, Xiugi et al. reported a gradual decrease in LDL-C levels accompanied by a deterioration in patients’ condition, and an inverse correlation between LDL-C and CRP levels [11].

There are various possible reasons for low LDL-C levels in COVID-19 positive patients. First, SARS-CoV-2-associated inflammation attenuates lipoprotein lipase (LPL) activity, thus interfering with LDL synthesis. Second, lipoproteins are susceptible to eradication by free radicals, levels of which significantly increase during infection, especially viral [11,66]. Third, researchers attribute low LDL-C levels to liver injury induced by COVID-19 [67], as well as to an increased uptake by monocyte-derived macrophages [68].

Whether LDL-C levels correlate with severity is still unclear as studies present contradictory results; therefore, further research is required. Studies by Hu et al., Wei et al. and Abbas et al. demonstrated that HDLs might be a much more promising indicator of COVID-19 severity to help with patient evaluations [10,11,47].

## 5. Triglyceride Structure, Role, Metabolism and Concentration in Patients with COVID-19

Elevated triglycerides (TGs) are associated with increased cardiovascular risk as they are a component of atherogenic dyslipidemia, alongside decreased HDL-C levels [68,69,70]. TG transporters are triglyceride-rich lipoproteins (TRLs), which are very-low-density lipoproteins (VLDLs) synthesized in the liver and chylomicrons produced in the enterocytes [71]. Like the LDL particles, TRLs can be taken up by macrophages present in the arterial wall, and therefore contribute to the inflammatory process [69,70]. Contrary to LDLs, the process of TRLs being taken up by macrophages may occur regardless of the oxidative promotion [70]. Increased levels of TRLs, consistent with elevated levels of triglycerides, are linked with atherogenesis, and therefore atherosclerosis due to their pro-inflammatory properties [71,72,73]. Inflammation is an underlying pathological cause of various diseases, including both cardiovascular and infectious ones [72].

Similar to other acute infections, COVID-19 causes an imbalance in lipid regulation, including TG and TRLs concentrations [11,74]. The TG concentration in patients with CoV disease was higher than levels obtained from the control groups, yet there was a decrease observed as the disease progressed, which is summarized in Table 3 [11,48].

TG breakdown products activate the NF-κB pathway, increasing the expression of pro-inflammatory cytokines [75]. TG-rich particles in the serum increase local inflammation, activate the complement pathway, and promote endothelial dysfunction. A decrease in triglyceride levels may be associated with a deterioration in the nutritional status of patients with an acute infection. However, TG levels were significantly higher in non-surviving-severe patients compared to the surviving patients, and therefore elevated TG-C levels in COVID-19 patients may be considered a biomarker of uncontrolled inflammation and an indicator of an increased risk of death [48,76]. Elevated TGs positively correspond with higher levels of acute phase proteins, ferritin and elevated D-dimer [76]. Furthermore, the presence of atherogenic dyslipidemia before the infection is associated with a worse COVID-19 infection prognosis and may be a predictor of a severe course of the disease [77].

## 6. Lipid-Lowering Therapies in Service of COVID-19 Treatment

Hyperlipidemia, atherosclerosis and their clinical implications, such as ischemic heart disease remain a considerable health burden all over the world [78]. Lifestyle modification and changing dietary habits are the first-line recommendations for patients affected by dyslipidemia; however, they are not always easy to be permanently implemented into a patient’s life [78]. Lipid-lowering agents are one of the most important pharmacological agents of prevention in both primary and secondary vascular events [78]. Statins are currently the most common lipid-lowering drugs, mainly because of their availability and safety. They were introduced more than 30 years ago [79]. The main mechanism of action of these drugs is to inhibit the early-stage cholesterol synthesis enzyme, 3-hydroxy-3-methylglutaryl-CoA reductase (HMGCR) [14], and therefore deprive hepatocytes of cholesterol, leading to the upregulation of LDL receptors, increased cholesterol uptake by cells, and consequently, lower serum cholesterol levels [79]. Apart from lowering LDLs, they are believed to reduce TG-C levels and increase HDL-C levels. Statins present many beneficial properties, independent of their lipid-lowering qualities, called pleiotropic activities, including anti-inflammatory, antithrombotic and immunomodulatory, just to name a few [80]. It is due to these additional activities that statins have been previously examined as adjuvants in viral infections, such as H1N1 infection [81,82,83] with promising results; therefore, the use of statins in COVID-19 is a matter worth our attention.

The potential benefits from using statins in COVID-19 are manyfold. Statins reduce CRP levels via the reduction of ox-LDL production [84], thereby alleviating inflammation. They improve inflamed vascular walls, thus preventing vascular complications such as myocardial infarction [85]. Moreover, by lowering membrane-bound cholesterol, statins significantly decrease the number of viral entry points according to Tang et al. [21].

Recent studies have shown that statins indeed mitigate the risk of acute respiratory distress syndrome and improve patient outcomes of pneumonia in general [86]. Makris et al. demonstrated significant improvements in survivability of patients with ventilator-associated pneumonia after introducing statins into therapy [86]. On the other hand, a different study found no such evidence in a similar trial [87]. A study conducted by Vahedian-Azimi et al. provided interesting results which indicated that there was a greater reduction in mortality observed in patients who were given statins during hospitalization for COVID-19 than in those previously treated with the drug [87]. There is also a discussion regarding which statin is the most efficient as an additional COVID-19 therapy. Some of them, such as atorvastatin, simvastatin and lovastatin are metabolized through the same paths as remdesivir; therefore, simultaneous use of these drugs is contraindicated [88,89]. In addition, in COVID-19 patients who already present with hypolipidemia, the reduction in LDL-C levels resulting from statin use may not have a desirable effect; therefore, further research is needed.

Additional lipid-lowering therapies targeting other lipid-metabolism pathways for individuals who do not tolerate or benefit from statins are: ezetimibe, an oral agent which inhibits the absorption of cholesterol in the intestines; as well as drugs inhibiting proprotein convertase subtilisin/kexin type 9 (PCSK9) in the form of monoclonal antibodies alirocumab and evolocumab, or as small molecule inhibitors [78,90]. PCSK9 inhibitors are relatively new drugs that strongly decrease LDL-C levels. Their mechanism of action inhibits circulating PCSK9 protein, thereby blocking degradation of the LDL receptor (LDLR), increasing its expression on the surface of hepatocytes and the uptake of LDL into the liver [90]. This injectable lipid-lowering therapy is dedicated especially for those with familial hypercholesterolemia (FH) [90,91,92]. FH is a genetic condition that causes significant increases in serum cholesterol and LDL-C levels. FH begins at a noticeably young age and responds poorly to oral therapy, and therefore represents an increased cardiovascular risk for individuals affected [92].

PCSK9 inhibitors are believed to exhibit anti-inflammatory properties thanks to down-regulating LDL-C levels. Hyperinflammation is one of COVID-19 characteristics and muting the excessive immune response may be beneficial in disease management [92]. COVID-19 patients that previously presented with cardiovascular disease were at a greater risk of a more severe course of the disease, COVID-19-induced cardiac injury, and therefore death [92]. As PCSK9 inhibitors have a positive impact on endothelial function they may have a role in the prevention of acute vascular incidents during COVID-19 and improve patients’ prognosis [91]. However, studies suggest that the beneficial role of PCSK9 inhibitors is not limited to the efficient lowering of LDL levels. PCSK9 molecules inhibit cellular interferon expression as they interact with the activating transcription factor 2 (ATF-2), so inhibiting PCSK9 counteracts this effect [93]. Studies focusing on the dengue virus that also used cholesterol as a proviral factor, suggested that PCSK9 inhibitors can be beneficial as adjunctive therapy. The dengue virus is believed to induce cellular PCSK9 expression as elevated serum PCSK9 levels were found in viremic dengue fever patients [93]. Viremia-induced increases in PCSK9 concentrations also report a notable decrease in type I interferon production [91,93]. What is more, the latest COVID-19 studies have implicated that defective type I interferon is a risk factor for severe viral pneumonia [91]. To conclude, implementing PCSK9 inhibitors may prevent the reduction in the antiviral genes’ expression, especially type I interferon; therefore, preventing the development of severe COVID-19 [91,92]. There were no interactions detected between PCSK9 inhibitors and antiviral drugs [92].

The hypolipidemic agents that affect SARS-CoV-2 infection, including fibrates and omega-3 polyunsaturated fatty acids (n3-PUFAs) have been studied. Among fibrates, drugs that are peroxisome proliferator-activated receptor alpha (PPAR-a) agonists, and thus induce lipoprotein lipase activity to reduce TGs and LDL serum levels, the COVID-19 infection modulating properties are credited to fenofibrate [94]. Fenofibrate has various pleiotropic effects which are anti-inflammatory, antioxidant and anti-angiogenic. These properties are useful in reducing the severity of COVID-19 as they help to modulate cytokine release during the disease [85]. In cellular models, fenofibrate was shown to reverse the viral-induced metabolic disturbances and inhibited the receptor binding domain for ACE2, and therefore prevented the infection [95]. Considering these findings and the relatively safe drug profile, fenofibrate may become an effective tool in the treatment of COVID-19. N3-PUFAs, including eicosapentaenoic acid (EPA), docosahexaenoic acid (DHA), and α-linoleic acid (ALA) are widely recognized for their beneficial effect on cardiovascular diseases. Diets rich in n3-PUFAs are linked with a lower risk of atherosclerosis which results from their hypolipidemic and anti-inflammatory properties [96]. Their hypolipidemic effect is linked to a reduction in serum triglyceride levels [96]. In the management of COVID-19, the anti-inflammatory properties of n3-PUFAs are used. Studies indicate that n3-PUFAs can regulate immune function by blocking hyperinflammatory reactions, and amplifying anti-inflammatory processes; therefore, reducing the incidence of systemic inflammatory response syndrome (SIRS) and multiple organ dysfunction syndrome (MODS) [97]. In a study targeting COVID-19 patients conducted by Doaei et al., patients treated with n3-PUFAs had significantly higher 1-month survival rates, reduced incidents of acidosis and metabolic complications compared to the control group without the supplementation [98]. Although not meeting the criteria for classical statistical significance, the results from a study conducted by Asher et al. also suggested that there was a lower COVID-19 mortality rate among patients with n3-PUFAs supplementation. [99] Moreover, according to the European Society for Parenteral and Enteral Nutrition expert statement, the use of omega-3 fatty acids may improve oxygenation in COVID-19 patients [100]. Nevertheless, further research is needed to precisely determine the role of these substances in COVID-19 treatment.

The introduction of lipid-lowering agents in therapies for patients with COVID-19 is promising as there are many theories and studies to support this idea. However, as divided as these studies are, they are all coherent in their statement that patients with COVID-19 who are already treated with statins should retain this treatment. As for the patients affected with COVID-19 without previous treatment with statins, despite the results of the study by Vahedian-Azimi et al. [89], these drugs should not be added to their therapy [13,14,21] until more extensive research has been conducted. The results on fenofibrate and n3-PUFA in COVID-19 are promising, although further studies are needed on these topics. PCSK9 inhibitors are believed to have a dual positive effect on SARS-CoV-2 infection through their lipid-lowering properties and by promoting the expression of type I interferon genes [91]. Researchers have suggested implementing a single dose to FH patients diagnosed with COVID-19 to improve their prognosis and, hopefully, prevent them from developing severe symptoms or complications [91,92]. Further research is necessary to test the clinical value of this treatment.

## 7. Conclusions

Due to the outbreak of COVID-19 pandemic, the entire world has faced many difficulties both in the health sector and in other areas of life. Despite its name, SARS-CoV-2 disrupts not only respiratory processes but can also cause systemic havoc. SARS-CoV-2 infection can present itself with gastrointestinal, dermatological, cardiovascular, as well as neurological and psychiatric symptoms. In our study, we focused on the impact of SARS-CoV-2 infection on lipid metabolism. The studies that we analyzed showed a decrease in serum cholesterol, HDL-C, LDL-C levels and TG anomalies during the infection. There was also a direct correlation between a decrease in these factors and the stage of the disease. This data support the validity of using these laboratory findings as markers of the severity of COVID-19 disease and its progression. However, both low and extremely high HDL-C levels, as well as abnormally high LDL-C and TG levels before the onset of the disease predisposes a severe course of the infection, and therefore is associated with a greater risk of death. Patients with a history of any oral lipid-lowering therapies, especially statins due to their pleiotropic features, should continue hypolipidemic treatment as it may play an adjunctive role in treating COVID-19 and diminishing complications of the disease. Despite the benefits mentioned, experts agree that statins should not be implemented into the treatment of individuals who have not previously received such medication. Research focused on the potential use of PCSK9 inhibitors as an adjuvant therapy in COVID-19 patients has given promising results. This lipid-lowering drug may be beneficial through reducing serum LDL levels; therefore, muting the hyperinflammatory state, and decreasing the risk of COVID-19-induced cardiovascular incidents and their fatal outcomes. Further research is needed to verify the clinical value of injecting PCSK9 inhibitors into patients with severe hyperlipidemia to assess whether they improve their prognosis. Studies on other hypolipemic agents, such as fenofibrate and n3-PUFA have given promising results, yet more research is needed to assess their role in disease management. Further research is also necessary to provide more information on the role of lipoproteins in the pathogenesis of SARS-CoV-2 infection, the direct mechanism of laboratory abnormalities, and the potential use of statins to optimize COVID-19 treatment.

## Figures and Tables

**Table 1:** Human plasma lipoproteins [10,11,14,15].

Human Plasma Lipoprotein Fractions and Their Basic Functions			
High triglyceride content		High in cholesterol	
Chylomicrons	VLDL	LDL	HDL
Transportation of lipids from the small intestine to hepatic and muscle tissues, delivering triglycerides to adipose tissue	Transportation of triglycerides synthesized in the liver to adipose and muscle tissues	Delivering cholesterol molecules from the liver to cells	Reverse cholesterol transport—removal of excess cholesterol from cells and transporting it to the liver

**Table 2:** Changes in HDL levels in patients with COVID-19 compared to healthy patients.

Author	Number of COVID Patients	HDL-C in COVID-19 Patients, MEDIAN, [mmol/L]	HDL-C in Control Group, MEDIAN, [mmol/L]	*p*-Value	HDLs in Non-severe COVID Patients, MEDIAN, [mmol/L]	HDLs in Severe COVID Patients, MEDIAN, [mmol/L]	*p*-Value
Mohammedsaeed et al. [45]	80	1.05	2.9	0.04	-	-	-
Wang et al. [47]	228	0.79	1.37	<0.001	0.79	0.69	0.0032
Huang et al. [46]	218	1.02	1.52	<0.05	1.15	0.83	<0.05
Hu et al. [43]	114	1.08	1.27	<0.001	1.21	1.01	<0.001

**Table 3:** Level of triglycerides in COVID-19 patients in relation to the severity of the disease.

	Levels of TG in COVID-19 Patients ^1^	
	Mild COVID	Severe COVID
Wei et al. [11]	1.69 (1.4, 2.4)	1.6 (1.0, 2.13)
Sun et al. [44]	1.21 (0.81, 1.80)	0.96 (0.70, 1.62)
Masana et al. [61]	1.61 (1.13, 2.18)	1.94 (1.39, 2.88)

1—Values presented are median (outliers) (mmol/L).

## Data Availability

Data is contained within the article.

## References

[1] Wang C., Horby P.W., Hayden F.G., Gao G.F. (2020). A novel coronavirus outbreak of global health concern. Lancet.

[2] Yang P., Wang X. (2020). COVID-19: A new challenge for human beings. Cell. Mol. Immunol..

[3] Khan M., Adil S.F., Alkhathlan H.Z., Tahir M.N., Saif S., Khan M., Khan S.T. (2020). COVID-19: A Global Challenge with Old History, Epidemiology and Progress So Far. Molecules.

[4] Ophinni Y., Hasibuan A.S., Widhani A., Maria S., Koesnoe S., Yunihastuti E., Karjadi T.H., Rengganis I., Djauzi S. (2020). COVID-19 Vaccines: Current Status and Implication for Use in Indonesia. Acta Med. Indones..

[5] Lazarus J.V., Ratzan S.C., Palayew A., Gostin L.O., Larson H.J., Rabin K., Kimball S., El-Mohandes A. (2021). A global survey of potential acceptance of a COVID-19 vaccine. Nat. Med..

[6] Akarsu B., Canbay Özdemir D., Ayhan Baser D., Aksoy H., Fidancı İ., Cankurtaran M. (2021). While studies on COVID-19 vaccine is ongoing, the public’s thoughts and attitudes to the future COVID-19 vaccine. Int. J. Clin. Pract..

[7] Binagwaho A., Mathewos K., Davis S. (2021). Equitable and Effective Distribution of the COVID-19 Vaccines—A Scientific and Moral Obligation. Int. J. Health Policy Manag..

[8] Li T., Huang T., Guo C., Wang A., Shi X., Mo X., Lu Q., Sun J., Hui T., Tian G. (2021). Genomic variation, origin tracing, and vaccine development of SARS-CoV-2: A systematic review. Innovation.

[9] Behzad S., Aghaghazvini L., Radmard A.R., Gholamrezanezhad A. (2020). Extrapulmonary manifestations of COVID-19: Radiologic and clinical overview. Clin. Imaging.

[10] Rezaei A., Neshat S., Heshmat-Ghahdarijani K. (2021). Alterations of Lipid Profile in COVID-19: A Narrative Review. Curr. Probl. Cardiol..

[11] Wei X., Zeng W., Su J., Wan H., Yu X., Cao X., Tan W., Wang H. (2020). Hypolipidemia is associated with the severity of COVID-19. J. Clin. Lipidol..

[12] Abu-Farha M., Thanaraj T.A., Qaddoumi M.G., Hashem A., Abubaker J., Al-Mulla F. (2020). The Role of Lipid Metabolism in COVID-19 Virus Infection and as a Drug Target. Int. J. Mol. Sci..

[13] Radenkovic D., Chawla S., Pirro M., Sahebkar A., Banach M. (2020). Cholesterol in Relation to COVID-19: Should We Care about It?. J. Clin. Med..

[14] Kočar E., Režen T., Rozman D. (2021). Cholesterol, lipoproteins, and COVID-19: Basic concepts and clinical applications. Biochim. Biophys. Acta Mol. Cell Biol. Lipids.

[15] Meilhac O., Tanaka S., Couret D. (2020). High-Density Lipoproteins Are Bug Scavengers. Biomolecules.

[16] Wei C., Wan L., Zhang Y., Fan C., Yan Q., Yang X., Gong J., Yang H., Li H., Zhang J. (2020). Cholesterol Metabolism-Impact for SARS-CoV-2 Infection Prognosis, Entry, and Antiviral Therapies. MedRxiv.

[17] Baglivo M., Baronio M., Natalini G., Beccari T., Chiurazzi P., Fulcheri E., Petralia P.P., Michelini S., Fiorentini G., Miggiano G.A. (2020). Natural small molecules as inhibitors of coronavirus lipid-dependent attachment to host cells: A possible strategy for reducing SARS-COV-2 infectivity?. Acta Biomed..

[18] Jeon J.H., Lee C. (2018). Cholesterol is important for the entry process of porcine deltacoronavirus. Arch. Virol..

[19] Li G., Li Y., Yamate M., Li S., Ikuta K. (2007). Lipid rafts play an important role in the early stage of severe acute respiratory syndrome-coronavirus life cycle. Microbes Infect..

[20] Hoffmann M., Kleine-Weber H., Schroeder S., Krüger N., Herrler T., Erichsen S., Schiergens T.S., Herrler G., Wu N.-H., Nitsche A. (2020). SARS-CoV-2 Cell Entry Depends on ACE2 and TMPRSS2 and Is Blocked by a Clinically Proven Protease Inhibitor. Cell.

[21] Tang Y., Hu L., Liu Y., Zhou B., Qin X., Ye J., Shen M., Wu Z., Zhang P. (2021). Possible mechanisms of cholesterol elevation aggravating COVID-19. Int. J. Med. Sci..

[22] Glende J., Schwegmann-Wessels C., Al-Falah M., Pfefferle S., Qu X., Deng H., Drosten C., Naim H.Y., Herrler G. (2008). Importance of cholesterol-rich membrane microdomains in the interaction of the S protein of SARS-coronavirus with the cellular receptor angiotensin-converting enzyme 2. Virology.

[23] Wang H., Yuan Z., Pavel M.A., Hansen S.B. (2021). The role of high cholesterol in age-related COVID19 lethality. bioRxiv.

[24] Meher G., Bhattacharjya S., Chakraborty H. (2019). Membrane Cholesterol Modulates Oligomeric Status and Peptide-Membrane Interaction of Severe Acute Respiratory Syndrome Coronavirus Fusion Peptide. J. Phys. Chem. B.

[25] Sahebkar A., Kotani K., Serban C., Ursoniu S., Mikhailidis D.P., Jones S.R., Ray K.K., Blaha M.J., Rysz J., Toth P.P. (2015). Statin therapy reduces plasma endothelin-1 concentrations: A meta-analysis of 15 randomized controlled trials. Atherosclerosis.

[26] Bianconi V., Sahebkar A., Banach M., Pirro M. (2017). Statins, haemostatic factors and thrombotic risk. Curr. Opin. Cardiol..

[27] Hu X., Chen D., Wu L., He G., Ye W. (2020). Low serum cholesterol level among patients with COVID-19 infection in Wenzhou, China. SSRN Electr. J..

[28] Marcello A., Civra A., Milan Bonotto R., Alves L.N., Rajasekharan S., Giacobone C., Caccia C., Cavalli R., Adami M., Brambilla P. (2020). The cholesterol metabolite 27-hydroxycholesterol inhibits SARS-CoV-2 and is markedly decreased in COVID-19 patients. Redox Biol..

[29] Ghzaiel I., Sassi K., Zarrouk A., Nury T., Ksila M., Leoni V., Bouhaouala-Zahar B., Hammami S., Hammami M., Mackrill J.J. (2021). 7-Ketocholesterol: Effects on viral infections and hypothetical contribution in COVID-19. J. Steroid Biochem. Mol. Biol..

[30] Xu Y., Wang D., Liu H., Ding X., Zhang X., Liu S., Sun T. (2022). Prognostic value of PCSK9 and blood lipid in patients with sepsis. Zhonghua Wei Zhong Bing Ji Jiu Yi Xue.

[31] Reisinger A.C., Schuller M., Sourij H., Stadler J.T., Hackl G., Eller P., Marsche G. (2022). Impact of Sepsis on High-Density Lipoprotein Metabolism. Front. Cell Dev. Biol..

[32] Cirstea M., Walley K.R., Russell J.A., Brunham L.R., Genga K.R., Boyd J.H. (2017). Decreased high-density lipoprotein cholesterol level is an early prognostic marker for organ dysfunction and death in patients with suspected sepsis. J. Crit. Care.

[33] Ben-Aicha S., Badimon L., Vilahur G. (2020). Advances in HDL: Much More than Lipid Transporters. Int. J. Mol. Sci..

[34] Sulaiman W.N., Caslake M.J., Delles C., Karlsson H., Mulder M.T., Graham D., Freeman D.J. (2016). Does high-density lipoprotein protect vascular function in healthy pregnancy?. Clin. Sci..

[35] Barter P.J., Nicholls S., Rye K.A., Anantharamaiah G.M., Navab M., Fogelman A.M. (2004). Antiinflammatory properties of HDL. Circ. Res..

[36] Baker J., Ayenew W., Quick H., Hullsiek K.H., Tracy R., Henry K., Duprez D., Neaton J.D. (2010). High-density lipoprotein particles and markers of inflammation and thrombotic activity in patients with untreated HIV infection. J. Infect. Dis..

[37] Florentin M., Liberopoulos E.N., Wierzbicki A.S., Mikhailidis D.P. (2008). Multiple actions of high-density lipoprotein. Curr. Opin. Cardiol..

[38] Mineo C., Deguchi H., Griffin J.H., Shaul P.W. (2006). Endothelial and antithrombotic actions of HDL. Circ. Res..

[39] Khovidhunkit W., Kim M.S., Memon R.A., Shigenaga J.K., Moser A.H., Feingold K.R., Grunfeld C. (2004). Effects of infection and inflammation on lipid and lipoprotein metabolism: Mechanisms and consequences to the host. J. Lipid Res..

[40] Heinecke J.W. (2020). Does HDL (High-Density Lipoprotein) Play a Causal Role in Host Defenses Against Infection?. Arterioscler. Thromb. Vasc. Biol..

[41] Srinivas R.V., Venkatachalapathi Y.V., Rui Z., Owens R.J., Gupta K.B., Srinivas S.K., Anantharamaiah G.M., Segrest J.P., Compans R.W. (1991). Inhibition of virus-induced cell fusion by apolipoprotein A-I and its amphipathic peptide analogs. J. Cell. Biochem..

[42] Gordon S.M., Hofmann S., Askew D.S., Davidson W.S. (2011). High density lipoprotein: It’s not just about lipid transport anymore. Trends Endocrinol. Metab..

[43] Roca B., Mendoza M.A., Roca M. (2020). Within subject variability of HDL-cholesterol in HIV-infected patients. Postgrad. Med..

[44] Cao W.J., Wang T.T., Gao Y.F., Wang Y.Q., Bao T., Zou G.Z. (2019). Serum Lipid Metabolic Derangement is Associated with Disease Progression During Chronic HBV Infection. Clin. Lab..

[45] Pirillo A., Catapano A.L., Norata G.D. (2015). HDL in Infectious Diseases and Sepsis. High Density Lipoproteins.

[46] van Leeuwen H.J., Heezius E.C., Dallinga G.M., van Strijp J.A., Verhoef J., van Kessel K.P. (2003). Lipoprotein metabolism in patients with severe sepsis. Crit. Care Med..

[47] Tanaka S., Diallo D., Delbosc S., Genève C., Zappella N., Yong-Sang J., Patche J., Harrois A., Hamada S., Denamur E. (2019). High-density lipoprotein (HDL) particle size and concentration changes in septic shock patients. Ann. Intensive Care.

[48] Tanaka S., Labreuche J., Drumez E., Harrois A., Hamada S., Vigué B., Couret D., Duranteau J., Meilhac O. (2017). Low HDL levels in sepsis versus trauma patients in intensive care unit. Ann. Intensive Care.

[49] Hu X., Chen D., Wu L., He G., Ye W. (2020). Declined serum high density lipoprotein cholesterol is associated with the severity of COVID-19 infection. Clin. Chim. Acta.

[50] Sun J.T., Chen Z., Nie P., Ge H., Shen L., Yang F., Qu X.L., Ying X.Y., Zhou Y., Wang W. (2020). Lipid Profile Features and Their Associations with Disease Severity and Mortality in Patients With COVID-19. Front. Cardiovasc. Med..

[51] Mohammedsaeed W., Alahamadey Z.Z., Khan M. (2020). Alteration of lipid profile in COVID-19 Saudi patients at Al-Madinah Al-Munawarah. Infection.

[52] Wendel M., Paul R., Heller A.R. (2007). Lipoproteins in inflammation and sepsis. II. Clinical aspects. Intensive Care Med..

[53] Chidambaram V., Shanmugavel Geetha H., Kumar A., Majella M.G., Sivakumar R.K., Voruganti D., Mehta J.L., Karakousis P.C. (2022). Association of Lipid Levels With COVID-19 Infection, Disease Severity and Mortality: A Systematic Review and Meta-Analysis. Front. Cardiovasc. Med..

[54] Ettinger W.H., Harris T., Verdery R.B., Tracy R., Kouba E. (1995). Evidence for inflammation as a cause of hypocholesterolemia in older people. J. Am. Geriatr. Soc..

[55] Wei C., Wan L., Yan Q., Wang X., Zhang J., Yang X., Zhang Y., Fan C., Li D., Deng Y. (2020). HDL-scavenger receptor B type 1 facilitates SARS-CoV-2 entry. Nat. Metab..

[56] Huang S., Zhou C., Yuan Z., Xiao H., Wu X. (2021). The clinical value of high-density lipoprotein in the evaluation of new coronavirus pneumonia. Adv. Clin. Exp. Med..

[57] Wang G., Deng J., Li J., Wu C., Dong H., Wu S., Zhong Y. (2021). The Role of High-Density Lipoprotein in COVID-19. Front. Pharmacol..

[58] Shen B., Yi X., Sun Y., Bi X., Du J., Zhang C., Quan S., Zhang F., Sun R., Qian L. (2020). Proteomic and Metabolomic Characterization of COVID-19 Patient Sera. Cell.

[59] Begue F., Tanaka S., Mouktadi Z., Rondeau P., Veeren B., Diotel N., Tran-Dinh A., Robert T., Vélia E., Mavingui P. (2021). Altered high-density lipoprotein composition and functions during severe COVID-19. Sci. Rep..

[60] Aung N., Khanji M.Y., Munroe P.B., Petersen S.E. (2020). Causal Inference for Genetic Obesity, Cardiometabolic Profile and COVID-19 Susceptibility: A Mendelian Randomization Study. Front. Genet..

[61] Madsen C.M., Varbo A., Tybjærg-Hansen A., Frikke-Schmidt R., Nordestgaard B.G. (2018). U-shaped relationship of HDL and risk of infectious disease: Two prospective population-based cohort studies. Eur. Heart J..

[62] Feingold K.R., Anawalt B., Boyce A., Chrousos G., de Herder W.W., Dhatariya K., Dungan K., Grossman A., Hershman J.M., Hofland J. (2000). Introduction to Lipids and Lipoproteins. Endotext.

[63] Young S.G., Parthasarathy S. (1994). Why are low-density lipoproteins atherogenic?. West. J. Med..

[64] Biswas H.H., Gordon A., Nuñez A., Perez M.A., Balmaseda A., Harris E. (2015). Lower Low-Density Lipoprotein Cholesterol Levels Are Associated with Severe Dengue Outcome. PLoS Negl. Trop. Dis..

[65] Marin-Palma D., Sirois C.M., Urcuqui-Inchima S., Hernandez J.C. (2019). Inflammatory status and severity of disease in dengue patients are associated with lipoprotein alterations. PLoS ONE.

[66] Osuna-Ramos J.F., Rendón-Aguilar H., Jesús-González L.A.D., Reyes-Ruiz J.M., Espinoza-Ortega A.M., Ochoa-Ramírez L.A. (2020). Serum lipid profile changes and their clinical diagnostic significance in COVID-19 Mexican Patients. medRxiv.

[67] Tanaka S., De Tymowski C., Assadi M., Zappella N., Jean-Baptiste S., Robert T., Peoc’h K., Lortat-Jacob B., Fontaine L., Bouzid D. (2020). Lipoprotein concentrations over time in the intensive care unit COVID-19 patients: Results from the ApoCOVID study. PLoS ONE.

[68] Zidar D.A., Juchnowski S., Ferrari B. (2015). Oxidized LDL levels are increased in HIV infection and may drive monocyte activation. JAIDS J. Acquir. Immune. Defic. Syndr..

[69] Chait A., Ginsberg H.N., Vaisar T., Heinecke J.W., Goldberg I.J., Bornfeldt K.E. (2020). Remnants of the Triglyceride-Rich Lipoproteins, Diabetes, and Cardiovascular Disease. Diabetes.

[70] Khetarpal S.A., Rader D.J. (2015). Triglyceride-rich lipoproteins and coronary artery disease risk: New insights from human genetics. Arterioscler. Thromb. Vasc. Biol..

[71] Budoff M. (2016). Triglycerides and Triglyceride-Rich Lipoproteins in the Causal Pathway of Cardiovascular Disease. Am. J. Cardiol..

[72] Rygiel K. (2018). Hypertriglyceridemia—Common Causes, Prevention and Treatment Strategies. Curr. Cardiol. Rev..

[73] Boden G. (2011). Obesity, insulin resistance and free fatty acids. Curr. Opin. Endocrinol. Diabetes Obes..

[74] Generoso G., Janovsky C.C.P.S., Bittencourt M.S. (2019). Triglycerides and triglyceride-rich lipoproteins in the development and progression of atherosclerosis. Curr. Opin. Endocrinol. Diabetes Obes..

[75] Gordillo-Moscoso A., Ruiz E., Carnero M., Reguillo F., Rodriguez E., Tejerina T., Redondo S. (2013). Relationship between serum levels of triglycerides and vascular inflammation, measured as COX-2, in arteries from diabetic patients: A translational study. Lipids Health Dis..

[76] Filippas-Ntekouan S., Liberopoulos E., Elisaf M. (2017). Lipid testing in infectious diseases: Possible role in diagnosis and prognosis. Infection.

[77] Masana L., Correig E., Ibarretxe D., Anoro E., Arroyo J.A., Jericó C., Guerrero C., Miret M., Näf S., Pardo A. (2021). STACOV-XULA research group. Low HDL and high triglycerides predict COVID-19 severity. Sci. Rep..

[78] Ray K.K., Corral P., Morales E., Nicholls S.J. (2019). Pharmacological lipid-modification therapies for prevention of ischaemic heart disease: Current and future options. Lancet.

[79] Stancu C., Sima A. (2001). Statins: Mechanism of action and effects. J. Cell Mol. Med..

[80] Oesterle A., Laufs U., Liao J.K. (2017). Pleiotropic Effects of Statins on the Cardiovascular System. Circ. Res..

[81] Lee K.C.H., Sewa D.W., Phua G.C. (2020). Potential role of statins in COVID-19. Int. J. Infect. Dis..

[82] Vandermeer M.L., Thomas A.R., Kamimoto L., Reingold A., Gershman K., Meek J., Farley M.M., Ryan P., Lynfield R., Baumbach J. (2012). Association between use of statins and mortality among patients hospitalized with laboratory-confirmed influenza virus infections: A multistate study. J. Infect. Dis..

[83] Fedson D.S. (2013). Treating influenza with statins and other immunomodulatory agents. Antivir. Res..

[84] Fedson D.S., Opal S.M., Rordam O.M. (2020). Hiding in Plain Sight: An Approach to Treating Patients with Severe COVID-19 Infection. mBio.

[85] Hileman C.O., Turner R., Funderburg N.T., Semba R.D., McComsey G.A. (2016). Changes in oxidized lipids drive the improvement in monocyte activation and vascular disease after statin therapy in HIV. AIDS.

[86] Katsiki N., Banach M., Mikhailidis D.P. (2020). Lipid-lowering therapy and renin-angiotensin-aldosterone system inhibitors in the era of the COVID-19 pandemic. Arch. Med. Sci..

[87] Barkas F., Milionis H., Anastasiou G., Liberopoulos E. (2021). Statins and PCSK9 inhibitors: What is their role in coronavirus disease 2019?. Med. Hypotheses.

[88] Papazian L., Roch A., Charles P.E., Penot-Ragon C., Perrin G., Roulier P., Goutorbe P., Lefrant J.Y., Wiramus S., Jung B. (2013). Effect of statin therapy on mortality in patients with ventilator-associated pneumonia: A randomized clinical trial. JAMA.

[89] Vahedian-Azimi A., Mohammadi S.M., Heidari Beni F., Banach M., Guest P.C., Jamialahmadi T., Sahebkar A. (2021). Improved COVID-19 ICU admission and mortality outcomes following treatment with statins: A systematic review and meta-analysis. Arch. Med. Sci..

[90] Scicali R., Di Pino A., Piro S., Rabuazzo A.M., Purrello F. (2020). May statins and PCSK9 inhibitors be protective from COVID-19 in familial hypercholesterolemia subjects?. Nutr. Metab. Cardiovasc. Dis..

[91] Vuorio A., Kovanen P.T. (2021). PCSK9 inhibitors for COVID-19: An opportunity to enhance the antiviral action of interferon in patients with hypercholesterolaemia. J. Intern. Med..

[92] Khan M., Singh G.K., Abrar S., Ganeshan R., Morgan K., Harky A. (2021). Pharmacotherapeutic agents for the management of COVID-19 patients with preexisting cardiovascular disease. Expert. Opin. Pharmacother..

[93] Gan E.S., Tan H.C., Le D.H.T., Huynh T.T., Wills B., Seidah N.G., Ooi E.E., Yacoub S. (2020). Dengue virus induces PCSK9 expression to alter antiviral responses and disease outcomes. J. Clin. Investig..

[94] Yasmin F., Zeeshan M.H., Ullah I. (2022). The role of fenofibrate in the treatment of COVID-19. Ann. Med. Surg..

[95] Davies S., Mycroft-West C., Pagani I., Hill H., Chen Y., Karlsson R., Bagdonaite I., Guimond S.E., Stamataki Z., De Lima M.A. (2021). The hyperlipidaemic drug fenofibrate significantly reduces infection by SARS-CoV-2 in cell culture models. Front. Pharmacol..

[96] Watanabe Y., Tatsuno I. (2020). Prevention of Cardiovascular Events with Omega-3 Polyunsaturated Fatty Acids and the Mechanism Involved. J. Atheroscler. Thromb..

[97] Taha A.M., Shaarawy A.S., Omar M.M., Abouelmagd K., Shalma N.M., Alhashemi M., Ahmed H.M., Allam A.H., Abd-ElGawad M. (2022). Effect of Omega-3 fatty acids supplementation on serum level of C-reactive protein in patients with COVID-19: A systematic review and meta-analysis of randomized controlled trials. J. Transl. Med..

[98] Doaei S., Gholami S., Rastgoo S., Gholamalizadeh M., Bourbour F., Bagheri S.E., Samipoor F., Akbari M.E., Shadnoush M., Ghorat F. (2021). The effect of omega-3 fatty acid supplementation on clinical and biochemical parameters of critically ill patients with COVID-19: A randomized clinical trial. J. Transl. Med..

[99] Asher A., Tintle N.L., Myers M., Lockshon L., Bacareza H., Harris W.S. (2021). Blood omega-3 fatty acids and death from COVID-19: A pilot study. Prostaglandins Leukot Essent Fat. Acids..

[100] Barazzoni R., Bischoff S.C., Breda J., Wickramasinghe K., Krznaric Z., Nitzan D., Pirlich M., Singer P. (2020). ESPEN expert statements and practical guidance for nutritional management of individuals with SARS-CoV-2 infection. Clin. Nutr..

